# Role of the venus kinase receptor in the female reproductive physiology of the desert locust, *Schistocerca gregaria*

**DOI:** 10.1038/s41598-017-11434-3

**Published:** 2017-09-15

**Authors:** Cynthia Lenaerts, Jolien Palmans, Elisabeth Marchal, Rik Verdonck, Jozef Vanden Broeck

**Affiliations:** Molecular Developmental Physiology and Signal Transduction, KU Leuven, Naamsestraat 59, P.O. Box 02465, B-3000 Leuven, Belgium

## Abstract

Venus kinase receptors (VKR) are a subfamily of invertebrate receptor tyrosine kinases, which have only recently been discovered. They contain an intracellular tyrosine kinase domain and an extracellular Venus FlyTrap domain. VKRs have been functionally and pharmacologically characterized in only two invertebrate species, namely the human parasite *Schistosoma mansoni* and the mosquito *Aedes aegypti*, where they play a crucial role in oogenesis. Here, we report the characterization of a VKR in the desert locust, *Schistocerca gregaria*. We performed an in-depth profiling study of the *SgVKR* transcript levels in different tissues throughout the female adult stage. Using the RNA interference technique, the possible role of *SgVKR* was investigated. *SgVKR* knockdown had significant effects on ovarian ecdysteroid levels and on the size of oocytes during the vitellogenic stage. *Sg*VKR is probably involved in the complex cross-talk between several important pathways regulating female reproductive physiology. Contrary to *A. aegypti* and *S. mansoni*, we cannot conclude that this receptor is essential for reproduction, since silencing *SgVKR* did not affect fecundity or fertility. Considering the evolutionary distance between *A. aegypti* and *S. gregaria*, as well as the differences in regulation of their female reproductive physiology, this article constitutes a valuable asset in better understanding VKRs.

## Introduction

In 2003, a novel receptor tyrosine kinase (RTK) was discovered in the human parasite, *Schistosoma mansoni* ^1^. This membrane receptor was designated as the venus kinase receptor (VKR), since it possesses an extracellular ligand-binding Venus FlyTrap (VFT) domain that is connected via a single membrane spanning segment with the intracellular tyrosine kinase (TK) domain. VKR-encoding genes have since then been identified in genomes of several other invertebrate species^2^. In most of these species, only one *vkr* gene was found, but the genomes of some Trematoda, such as *Schistosoma* ^3^, and Lepidoptera contain two *vkr* genes (*vkr1* and *vkr*2). It should also be noted that no *vkr* genes were found in two major invertebrate research models, *Drosophila melanogaster* and *Caenorhabditis elegans*. Phylogenetic analysis evinced that all putative VKRs are clustering as a monophyletic group and likely evolved from a common ancestor^2^.

The TK domain of VKR closely resembles that of insulin-like peptide receptors (InR) and contains all motifs that are crucial for tyrosine kinase activity, such as the ATP binding site (GxGxxG), the catalytic loop implicated in the phosphotransfer (HRDxAxRN), and the two putative autophosphorylation sites (YY)^4^. Using recombinant VKRs and *in vitro* kinase assays, it was demonstrated that VKRs are functional tyrosine kinase receptors that can form dimers and autophosphorylate^4,5^. Phylogenetic and structural analysis of the VFT module (VFTM) of VKRs and other VFTM-containing receptors resulted in the identification of a monophyletic VKR clade which is closely resembling the γ-aminobutyric acid type B receptors (GABA_B_R)^4^. VFTMs of class C G protein-coupled receptors (GPCRs), including GABA_B_R, bind small ligands such as Ca^2+^, GABA and other amino acid transmitters, and small sugars^6^. Class C GPCRs that bind amino acids contain a consensus motif of 8 residues in their VFTM, which can be found in most VKRs^2^. *In vitro* kinase assays have shown that the VKR of *Apis mellifera* and *Sm*VKR1 are activated by L-Arg, while *Sm*VKR2 is activated by Ca^2+^ ^3,4^. Moreover, for the amino acid recognition by VKRs the serine and arginine residues of the conserved 8 residue motif are required^2^. Very recently, the VKR of *Aedes aegypti* was also deorphanized and surprisingly *Aa*VKR was shown to bind to the rather ‘large’ ovary ecdysteroidogenic hormone (OEH)^5^. The *OEH* gene encodes a 149-residue preprohormone that is processed into an 86-residue peptide, which belongs to the neuroparsin family^7^.

Little is known about the role of VKRs, probably due to the absence of VKRs in *D. melanogaster*, *C. elegans* and vertebrates. Very recently, two papers were published implicating a role for VKRs in female reproductive physiology. Vanderstraete and coworkers^8^ have shown that *Sm*VKRs control female reproduction in *S. mansoni*, since RNA interference (RNAi)-mediated silencing of *SmVKR* resulted in disorganization of the ovary and defective egg formation. Also in *A. aegypti* VKR seems to be required for egg formation^5^. In mosquitoes, juvenile hormone (JH) is responsible for preparing the fat body for production of vitellogenin. After a blood meal, OEH stimulates ecdysteroid production by the ovaries, which in turn stimulates vitellogenin production by the fat body. RNAi-mediated knockdown of *AaVKR* resulted in significantly lower ecdysteroid and vitellogenin production, leading to disabled egg formation. Another proof of *AaVKR* being the OEH receptor, was the lack of vitellogenin biosynthesis in the dsVKR treated females upon injection of OEH^5^.

This chapter focuses on the role of *Sg*VKR in the adult female desert locust, *S. gregaria*. This ravenous swarm-forming phytophagous pest insect is a serious threat to the agricultural production in some of the world’s poorest countries, as swarms may contain thousands of millions of individuals eating everything on their path^9^. Contrary to *A. aegypti*, vitellogenin biosynthesis in *S. gregaria* is solely under the regulation of JH. Furthermore, neuroparsins act as anti-gonadotropic factors in *S. gregaria*, while in *A. aegypti* the neuroparsin-like OEH exerts a gonadotropic role. Given these differences in the regulation of female reproductive physiology between *A. aegypti* and *S. gregaria*, this study represents a valuable asset to better understand the role of the VKR subfamily throughout evolution. Here we report the cloning of *SgVKR*, as well as an in-depth tissue distribution of this receptor in adult female locusts, using qRT-PCR. Furthermore, using RNAi, we investigate the possible cross-talk of this receptor with several major hormonal pathways involved in female reproductive physiology, as well as its role in the maturation of oocytes, copulation behavior and post-copulation events.

## Materials and Methods

### Rearing of animals

The desert locusts, *S. gregaria*, were reared under crowded conditions (>200 locusts/cage) at constant temperature (32 ± 1 °C), constant day/night cycle (13:11 h photoperiod) and ambient relative humidity between 40% and 60%. The locusts were fed daily *ad libitum* with fresh cabbage leaves, supplemented with dry oat flakes. Following mating, females deposited their eggs in pots filled with a slightly moistened sand mixture (7 parts sand, 3 parts peat and 1 part water). Once a week these pots were collected and set apart in empty cages, where eggs were allowed to hatch into first instar larvae. In the described experiments, locusts were synchronized on the day of ecdysis into the adult stage. For the RNA interference experiments, locusts were injected one day after ecdysis, boost injections were given five, nine and thirteen days after ecdysis. Some locusts were dissected 12 days after ecdysis, while the others were observed for copulation behavior and post-copulation effects. Different experimental groups (distinctly labelled) were kept together in the same cage.

### Tissue collection

The locust tissues of interest were dissected under a binocular microscope and rinsed in locust Ringer solution (1 L: 8.766 g NaCl; 0.188 g CaCl_2_; 0.746 g KCl; 0.407 g MgCl_2_; 0.336 g NaHCO_3_; 30.807 g sucrose; 1.892 g trehalose). Tissues were immediately pooled in MagNA Lyser Green Beads Tubes (Roche) or RNase-free Screw Cap Microcentrifuge tubes and snap-frozen in liquid nitrogen to prevent RNA degradation. Tissues for the tissue and temporal expression profile of *SgVKR* were collected in three independent pools consisting of five or six animals each. For the RNA interference experiments, tissues were collected in five independent pools consisting of three animals each. Tissues were stored at −80 °C until further processing.

### RNA extraction and cDNA synthesis

Depending on the tissue, different RNA extraction methods were used. Brain and optic lobes, fat body, Malpighian tubules, male reproductive system (testes + accessory glands), female gonads (ovaria) and gut were transferred to MagNA Lyser Green Beads Tubes (Roche) and homogenized using a MagNa Lyser instrument (1 min, 6500 rpm; Roche). Subsequently, total RNA was extracted from these tissue homogenates using the RNeasy Lipid Tissue Kit (Qiagen) according to the manufacturer’s protocol. A DNase treatment (RNase-Free DNase set, Qiagen) was performed to eliminate potential genomic DNA contamination. Because of the relatively small size of the prothoracic glands (PG), corpora allata (CA) and corpora cardiaca (CC), and the ventral nerve cord (VNC), total RNA from these tissues was extracted using the RNAqueous-Micro Kit (Ambion) according to the manufacturer’s protocol. The manufacturer’s recommended DNase step was subsequently performed. Purity and concentration of the resulting RNA samples were checked using a Nanodrop spectrophotometer (Nanodrop ND-1000, Thermo Fisher Scientific, Inc.). For each RNA sample, cDNA was synthesized by reverse transcription of 500 ng of RNA with the PrimeScript™ RT reagent Kit (Perfect Real Time) (Takara, Invitrogen Life Technologies), using both random hexamer primers and oligo(dT) primers, according to the manufacturer’s protocol. The 10 µL reaction was diluted sixteen-fold with Milli-Q water (Millipore).

### Molecular cloning of *SgVKR*

The full length sequence for the *S. gregaria* orthologue of *VKR* was present in our in-house (unpublished) *S. gregaria* transcriptome database. The complete sequence was amplified by PCR using adult *S. gregaria* fat body cDNA of 12-day-old adult female locusts, primers (listed in Table 1) and Q5^®^ High-Fidelity DNA Polymerase (New England BioLabs^®^ Inc.). Following thermal cycling profile was applied: 94 °C for 3 min; 35 cycles of 94 °C for 30 s, 64 °C for 30 s and 72 °C for 2,5 min; 72 °C for 10 min. The amplification products were separated using horizontal agarose gel electrophoresis and visualized using UV. The band of the expected size was cut out and further purified using the GenElute™ Gel extraction Kit (Sigma-Aldrich Co.). The resulting DNA fragment was cloned into a pCR4-TOPO vector using the TOPO^®^ TA Cloning Kit (Invitrogen). Finally the sequence of the insert was confirmed using Sanger sequencing. The protein architecture was analyzed using the SMART software (http://smart.embl-heidelberg.de/).

**Table 1:** Oligonucleotide sequences for primers used in cloning and sequence analysis of *SgVKR*.

	**Forward primer**	**Reverse primer**
*SgVKR*	5′-CTCCAGGAGCAGGACATCAC-3′	5′-TCAAAGAATGGAGTCCACTTGCTTAATTC-3′

Abbreviations: *VKR = venus kinase receptor*.

### Quantitative real-time PCR (qRT-PCR)

Primers used in the qRT-PCR profiling are given in Table 2. These primers were validated by designing relative standard curves for gene transcripts with serial dilutions (5x) of female gonads, fat body or corpora allata/corpora cardiaca (CA/CC) complex cDNA. All qRT-PCR reactions were performed in duplicate in 96-well plates on a StepOne System (ABI Prism, Applied Biosystems). Each reaction contained 5 µL Fast SYBR^®^ Green Master Mix (Applied Biosystems), 0.5 µL Forward and Reverse primer (10 µM) and 4 µL previously diluted cDNA. Following thermal cycling profile was used: 95 °C for 10 min, followed by 40 cycles of 95 °C for 15 s and 60 °C for 60 s. Finally, a dissociation protocol was performed, allowing melt curve analysis to check for primer dimers. Only a single melting peak was found for all transcripts. Additionally, amplification products were analyzed using horizontal agarose gel electrophoresis and visualized using UV. Only a single band could be seen which was further cloned and sequenced (TOPO^®^ TA cloning kit for sequencing, Invitrogen) to confirm target specificity. Suitable reference genes were selected from a pool of candidate reference genes by means of the geNorm software^10,11^. “No template control” reactions confirmed there was no contamination. All qRT-PCR results were normalized to the relative quantity of the selected reference genes (indicated in figure legends) and calculated relative to the transcript level of a calibrator sample according to the comparative Ct method^10^. qRT-PCR was used to determine the tissue and temporal distribution of *SgVKR* in the adult stage. The cDNA samples were derived from adult female locusts, with exception of the male reproductive system. Moreover, qRT-PCR was performed to determine the knockdown efficiency of *SgVKR* as well as the effect on several genes of interest. GraphPad Prism 6 (GraphPad Software Inc.) was used to test the statistical significance of the observed differences for the RNAi experiments. Normalized relative quantities were log-transformed to allow the use of parametric statistical tests.

**Table 2:** Oligonucleotide sequences for primers used in qRT-PCR.

**Reference genes**	**Forward primer**	**Reverse primer**
**Target genes**	**Forward primer**	**Reverse primer**
*α-tubulin1A*	5′-TGACAATGAGGCCATCTATG-3′	5′-TGCTTCCATACCCAGGAATGA-3′
*CG13220*	5′-TGTTCAGTTTTGGCTCTGTTCTGA-3′	5′-ACTGTTCTCCGGCAGAATGC-3′
*Ubi*	5′-GACTTTGAGGTGTGGCGTAG-3′	5′-GGATCACAAACACAGAACGA-3′
*GAPDH*	5′-GTCTGATGACAACAGTGCAT-3′	5′-GTCCATCACGCCACAACTTTC-3′
*RP49*	5′-CGCTACAAGAAGCTTAAGAGGTCAT-3′	5′-CCTACGGCGCACTCTGTTG-3′
*β-actin*	5′-AATTACCATTGGTAACGAGCGATT-3′	5′-TGCTTCCATACCCAGGAATGA-3′
*EF1α*	5′-GATGCTCCAGGCCACAGAGA-3′	5′-TGCACAGTCGGCCTGTGAT-3′
*SgVKR*	5′-GCATCTTGGCATTGATTTGCTA-3′	5′-GGAATCTCCCATTTGTCAAGAGTT-3′
*SgSpo*	5′-CAACATCTTCACCAGCTACATGTG-3′	5′-GGGTCGTCGTAGTCGAAGGA-3′
*SgPhm*	5′-CGCAGAGCCCGGACAAC-3′	5′-CGAACATGTCGGCCATGA-3′
*SgDib*	5′-TAGCTGGAATGGACACAACATC-3′	5′-CTGGGTCTGGAAAATACTCTGG-3′
*SgSad*	5′-ATCGTGGCCGAGATTACGAA-3′	5′-AGCACCATCTCCGGATCCT-3′
*SgShd*	5′-CCGCCGTCATTGACTTCATA-3′	5′-GTGAGCTCCCAAGCGTGG-3′
*SgVg1*	5′-CCGCTGAACATCACTGCAAT-3′	5′-ACTTGGGCCAAATGGATGAG-3′
*SgVg2*	5′-GCTACCCGCAATCTGTAAAATACA-3′	5′-CGACTGTGAAAGGGCATTGA-3′
*SgKr-h1*	5′-CTCCAAGACGTTCATCCAGAG-3′	5′-TGCTTGGAGCAGGTGAAG-3′
*SgJHAMT*	5′-CGGAGCAAAGGCAAGCA-3′	5′-CCACTTCACCGCCTGGTTT-3′
*SgCYP15A1*	5′-AAAGCAACTTCATCATTCACAGATG-3′	5′-CAGAGCCAGCCATGAACAAA-3′

Abbreviations: *Ubi = ubiquitin conjugating enzyme 10, GAPDH = glyceraldehyde-3-phosphate dehydrogenase, RP49 = ribosomal protein 49, EF1α = elongation factor 1 alpha, VKR = venus kinase receptor, Spo = spook, Phm = phantom, Dib = disembodied, Sad = shadow, Shd = shade, Vg = vitellogenin, Kr-h1 = Krüppel-homolog 1, JHAMT = juvenile hormone acid methyltransferase, CYP15A1 = methyl farnesoate epoxidase.*

### RNA interference experiments

#### Production of dsRNA

dsRNA constructs for *SgVKR* were produced using the MEGAscript^®^ RNAi Kit (Ambion) according to the manufacturer’s protocol. This procedure is based on the high-yield transcription reaction of a user-provided linear transcript with a T7 promoter sequence. Forward and reverse primers flanked by the T7 promoter sequence were (Table 3) used in a PCR reaction with REDTaq^®^ DNA polymerase (Sigma-Aldrich Co.) to amplify a fragment of the target gene (453 or 530 bp). PCR products were analyzed using horizontal agarose gel electrophoresis and visualized using UV. Only a single band could be seen which was further cloned and sequenced (TOPO^®^ TA cloning kit for sequencing, Invitrogen) to confirm target specificity. Since only one band was observed after gel electrophoresis, the PCR product could directly be used in the RNA polymerase transcription reaction. The purity and concentration of the produced dsRNA was determined by means of spectrophotometry (Nanodrop ND-1000). To confirm dsRNA integrity, a small amount of the reaction product was checked on an agarose gel.

**Table 3:** Oligonucleotide sequences for primers used in dsRNA construct design. Underlined sequences are the T7 promoter sequences.

**Target genes**	**Forward primer**	**Reverse primer**
*SgVKR1*	5′-TAATACGACTCACTATAGGGAGAAAGGGTTGCTGCATTGACTG-3′	5′-TAATACGACTCACTATAGGGAGATCCACACTGCATCATAGGCA-3′
*SgVKR2*	5′-GAAATTAATACGACTCACTATAGGGCCCACTGTGTATGGAGGAGAA-3′	5′-GAAATTAATACGACTCACTATAGGGCCTCAAACATTGGCCTTGTC-3′
*GFP*	5′-TAATACGACTCACTATAGGGAGA AAGGTGATGCTACATACGGAA-3′	5′-TAATACGACTCACTATAGGGAGA ATCCCAGCAGCAGTTACAAAC-3′

Abbreviations: *VKR = venus kinase receptor, GFP = green fluorescent protein.*

#### RNAi experiment

Adult female locusts were injected in their abdomen one, five, nine and thirteen days after final ecdysis with 200 ng (in 10 µL Ringer solution) of dsRNA against *SgVKR*. Control locusts were injected with a *GFP* dsRNA construct (200 ng in 10 µL locust Ringer solution) following the same treatment plan. A first group of locusts was dissected 12 days after ecdysis to check the knockdown efficiency and the effect of the knockdown on the oocyte size, ecdysteroid titers in the hemolymph, ecdysteroid levels in the gonads, and the transcript levels of other genes of interest. A second group of locusts was used to observe copulation behavior and post-copulation events.

### Ecdysteroid measurements using an enzyme immunoassay (EIA)

Ecdysteroid titers in *S. gregaria* hemolymph and ecdysteroid levels in the female gonads were measured using an enzyme immunoassay, modified from Porcheron *et al*.^12^ and discussed by Pascual *et al*.^13^ and Lafont *et al*.^14^. In this protocol a peroxidase conjugate of 20-hydroxy-ecdysone (20E) was used as tracer together with rabbit L2 polyclonal antibodies. This L2 antiserum has a strong affinity for ecdysone (E), 3-deoxyecdysone and 2-deoxyecdysone and a 6- to 8-fold lower affinity for 20E. Both serum and tracer were very kindly given by Prof. J.P. Delbecque (Université de Bordeaux, France). Hemolymph samples were collected from 12-day-old adult female locusts by piercing the insect’s cuticle behind its hind leg and holding a capillary to the wound. 10 µL of hemolymph was collected from each animal, which was immediately transferred to 90 µL of cold ethanol (100%) and stored at −20 °C until further processing, as described by Marchal *et al*.^15^. For the sampling of the gonads, one ovarium was dissected from each 12-day-old adult female locust and transferred to 500 µL of cold ethanol (100%) and stored at −20 °C until further processing as described by Van Wielendaele *et al*.^16^. Both ‘free’ and total ecdysteroid levels were determined. ‘Free’ ecdysteroids are the non-conjugated ecdysteroids. The total ecdysteroid levels were determined after enzymatic conversion of the conjugated ecdysteroids in ‘free’ ecdysteroids. The standard curve used in all measurements was obtained with 20E, and therefore results are expressed as 20E equivalents. GraphPad Prism 6 (GraphPad Software Inc.) was used to test the statistical significance of the observed differences.

### Copulation behavior and post-copulation effects

To investigate the effect of the RNAi-mediated knockdown of *SgVKR* on the first display of copulation behavior and fertility, the locusts were injected as described in ‘RNA interference experiments’. Until day 8 control and experimental female locusts were kept with non-treated male locusts of the same age. As of day 8 female locusts were kept in individual small transparent cages. Starting on day 10 the females were combined daily with mature virgin male locusts and allowed to mate. When mating occurred, the male stayed another 22 hours with the female. When no mating was observed, the males were removed from the cage. We only observed actual copulation, i.e. attachment of the male and female genitalia. After mating the females were supplied with pots filled with a humid sand/turf mixture, necessary for oviposition. These pots were checked daily for egg pods and the total number of eggs per egg pod was counted (to analyze fecundity). Eggs were allowed to hatch by placing them on the humid sand/turf mixture and covering them with approximately 1 cm of this mixture. Hatching was observed daily and the total number of hatched larvae was counted in order to determine the percentage of hatchlings (to analyze fertility).

## Results

### Cloning and sequence analysis of *SgVKR*

**Figure 1:**
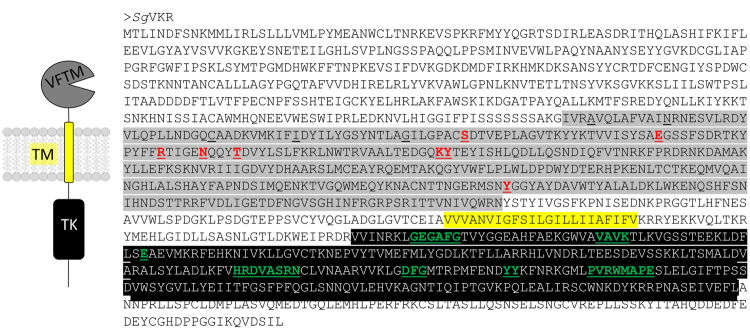
Sequence and structure of *Sg*VKR. *Left:* Schematic representation of a VKR, composed of different domains: VFTM = Venus FlyTrap module, TM = transmembrane domain and TK = tyrosine kinase domain (adapted from Ahier *et al*.)^4^. *Right:* Amino acid sequence of *Sg*VKR. The complete *SgVKR* sequence (GenBank acc. no. KY273096) was found in the in-house *S. gregaria* transcriptome database (our unpublished data) and was confirmed with PCR and Sanger sequencing. The VFTM is highlighted in grey, the TM domain is highlighted in yellow and the TK domain is highlighted in black. Underlined residues are conserved in most VKR sequences and those also in bold and red correspond to the amino-acid binding motif of VFTM^29^. The residues in green, bold and underlined are consensus sequences required for TK activity^17^.

The complete *Sg*VKR open reading frame (ORF) was found in the in-house *S. gregaria* transcriptome database and was fully confirmed by Sanger sequencing of the PCR amplicon acquired with the primers listed in Table 1. The *SgVKR* ORF (GenBank acc. no. KY273096) comprises 3834 nucleotides encoding a protein consisting of 1277 amino acids (Fig. 1). Based on similarities with previously predicted and identified VKRs^2,4^, we identified the typical VFTM and TK domains (Fig. 1, grey and black highlights). The VFTM contains the consensus motif of 8 residues (Fig. 1, red AA) that is known to participate in the binding of natural amino acids or their derivatives to class C GPCRs. Furthermore, the TK domain of *SgVKR* is composed of essential motifs (GxGxxG, VAVKx_16_E, HRDVxxRNxL, DFG, YY and PVRWMxPE) required for TK activity (Fig. 1, green AA)^17^.

### Transcript level studies

**Figure 2:**
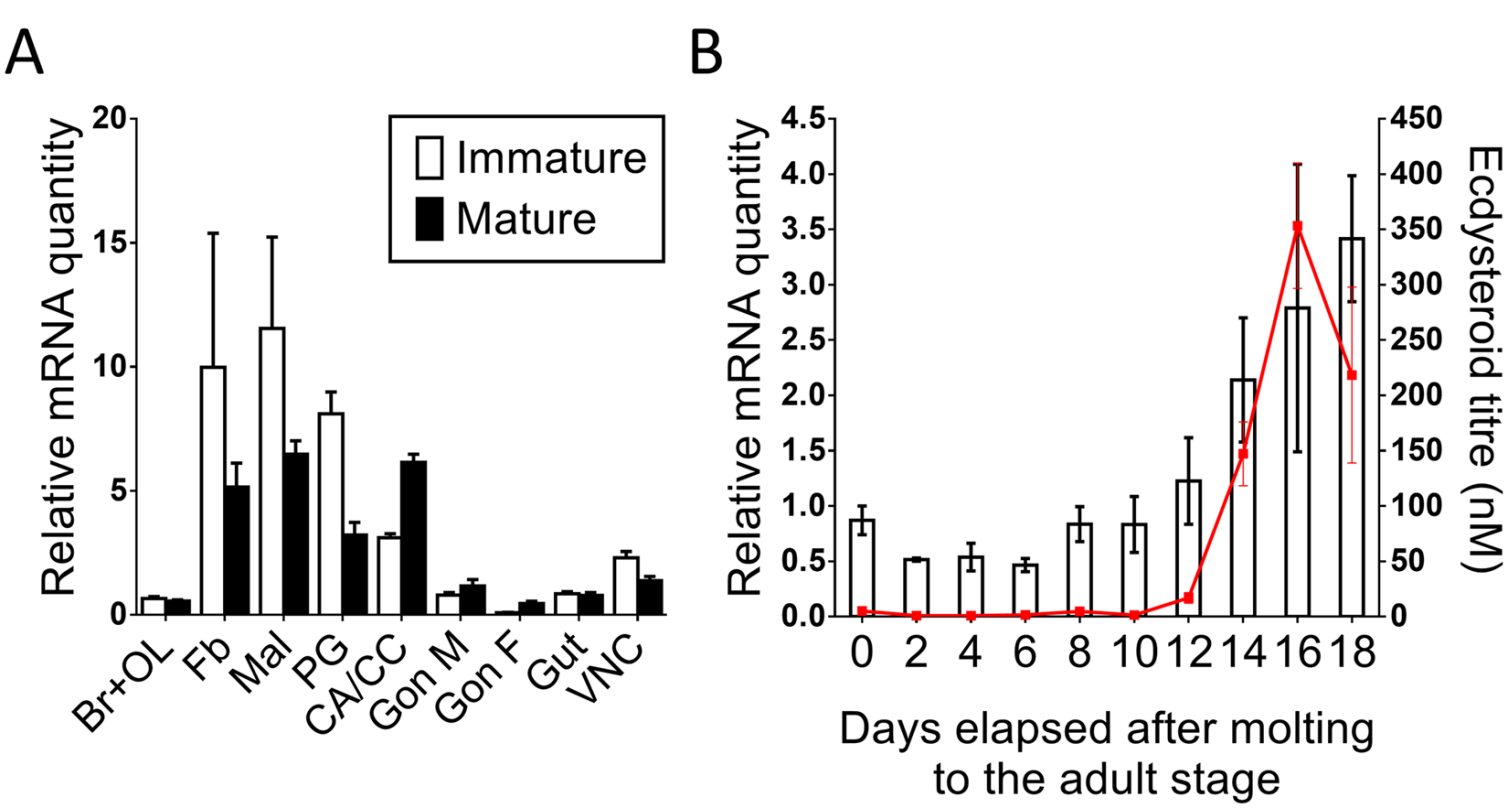
Tissue and temporal distribution of *SgVKR*. (**A**) Tissue distribution of *SgVKR* in immature (white) and mature (black) adult locusts. Relative transcript levels of *SgVKR* were measured in different adult tissues using qRT-PCR. All tissues, except the male reproductive system (Gon M), were dissected from immature, 3-day-old female locusts or mature female locusts with an oocyte size between 3 and 6 mm. The male reproductive system was dissected from immature, 3-day-old and mature, 12-day-old male locusts. The data represent mean ± S.E.M. of three independent pools of five animals, run in duplicate and normalized to α-*tubulin1A, CG13220* and *ubiquitin* transcript levels. Abbreviations X-axis: Br + OL: brain + optic lobes; Fb: fat body; Mal: Malpighian tubules; PG: prothoracic glands; CA + CC: corpora allata + corpora cardiaca; Gon M: male reproductive system (testes + accessory glands); Gon F: female gonads (ovaries); Gut: for-, mid- and hindgut; VNC: ventral nerve cord. (**B**) Temporal distribution profile of *SgVKR* in the female gonads during the first reproductive cycle. Using qRT-PCR, relative transcript levels of *SgVKR* were measured every other day in the female gonads, starting on the day of molting to the adult stage (day 0). Data represent mean ± S.E.M. of three independent pools of six animals, run in duplicate and normalized to *CG13220* and *ubiquitin* transcript levels. The ecdysteroid titre (red line), expressed in nM, throughout the first reproductive cycle was measured with an EIA. The data represent mean ± S.E.M. of 6-18 hemolymph samples taken from different animals.

Tissue and temporal transcript profiles of *SgVKR* were determined using qRT-PCR. The tissue distribution profile has been studied in immature and mature female tissues, as well as in the male reproductive system (Fig. 2A), while the temporal distribution profile has been studied in the female gonads, every other day throughout the first reproductive cycle (Fig. 2B).

The tissue distribution of *SgVKR* (Fig. 2A) shows a wide profile, with the highest transcript levels in the fat body, the Malpighian tubules, the prothoracic glands and the CA/CC complex. The transcript levels of *SgVKR* are significantly higher in the prothoracic glands and the ventral nerve cord of immature females compared to mature females, while they are significantly lower in the CA/CC complex and gonads of immature females compared to mature females.

Given the significantly higher expression of *SgVKR* in mature female gonads, and the fact that some VKRs play an important role in female reproductive physiology of other invertebrate species^5,8^, we investigated the temporal expression profile of the *SgVKR* transcript in the female gonads throughout the first reproductive cycle (Fig. 2B). We observed an increase in transcript levels towards the end of the first gonadotrophic cycle, suggesting a possible role for this receptor in locust reproduction.

### RNA interference of *SgVKR*

To investigate the role of *SgVKR* in the female reproductive physiology, the RNAi technique was used to silence *SgVKR*. To eliminate possible off-target effects two separate dsRNA constructs were designed for *SgVKR*, one in the VFTM and one in the TK domain. Similar results were obtained with both constructs. Female locusts were injected with 200 ng dsRNA against *SgVKR* or *GFP* (control) one, five, nine and thirteen days after molting to the adult stage. One group of locusts was sacrificed on day 12 to investigate the knockdown efficiency and the effect of the knockdown on the oocyte size, ecdysteroid biosynthesis and the transcript levels of several genes of interest. The other group of locusts was used to investigate the effect of the knockdown on copulation behavior and fertility.

### Knockdown efficiency

The knockdown efficiency of *SgVKR* was investigated in different tissues of 12-day-old adult female locusts, using qRT-PCR (Suppl. Figure 1). A reduction of 80%, 69% and 65% in relative mRNA levels of *SgVKR* could be measured in respectively the ovaries, the fat body and the CA/CC complex. From these significant reductions it can be concluded that *SgVKR* was successfully silenced.

### Effect on reproductive physiology

**Figure 3:**
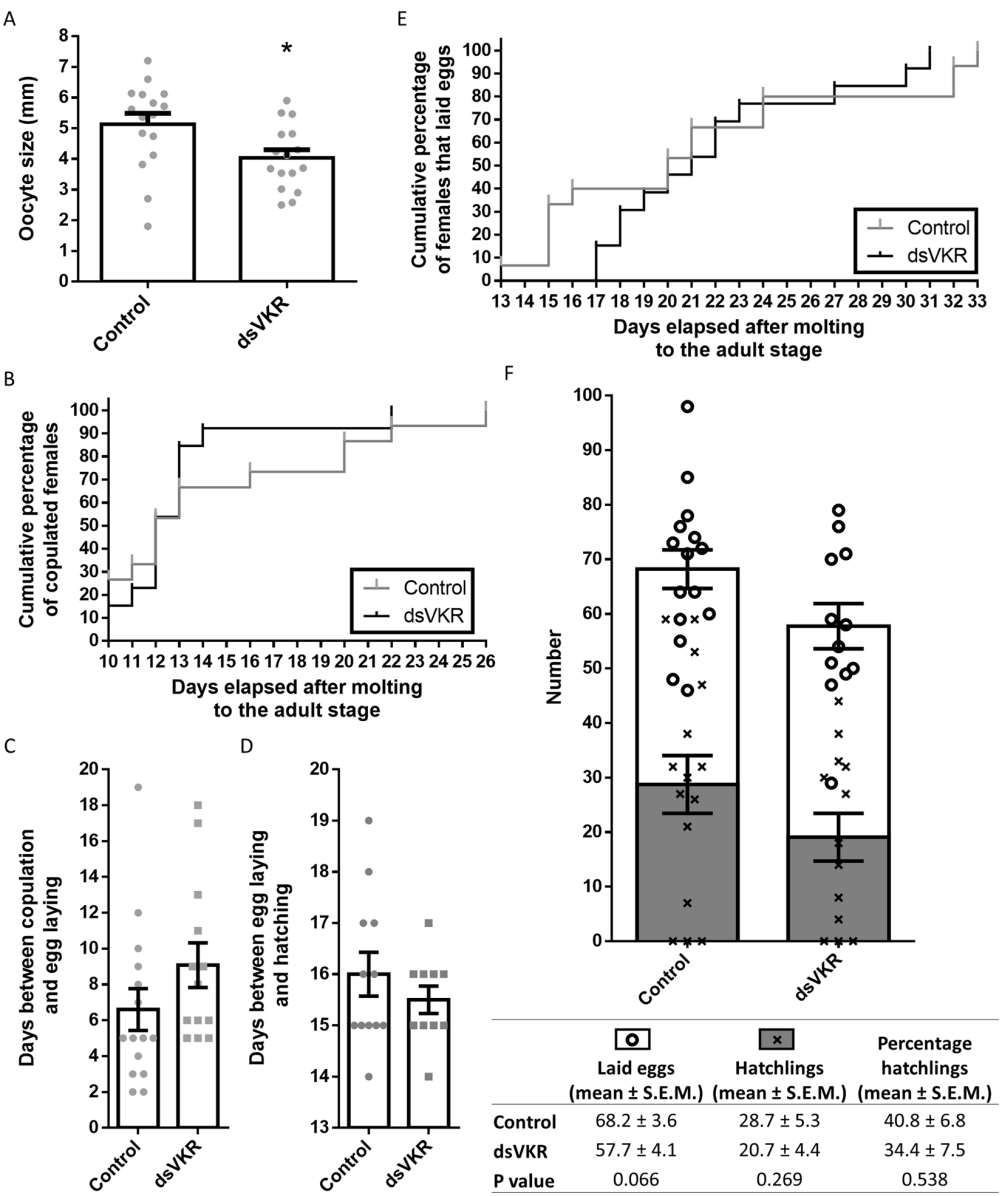
Effect of RNAi-mediated knockdown of *SgVKR* on the oocyte size in 12-day-old *S. gregaria* and on copulation behavior, fecundity and fertility. Locusts were injected with 200 ng of dsRNA against *SgVKR* or *GFP* (control) one, five, nine and thirteen days after molting to the adult stage. (**A**) For each 12-day-old female locust the average size of five oocytes was calculated. The data represent mean ± S.E.M. of the average oocyte sizes of sixteen individual locusts (bars), as well as the average oocyte size of the individual locusts (grey dots) (N = 16). The significant difference (p < 0.05) is indicated with an asterisk (*) (two-sided unpaired *t*-test). (**B**) Starting from day 10 of the adult stage the females were assayed for displaying mating behavior, by allowing them to mate with mature virgin males. The cumulative percentage of females that mated is presented (Log-rank (Mantel-Cox) test; N = 13-15; p-value = 0.5045). (**C-E**) Mated females were allowed to lay eggs. The days between copulation and egg laying (**C**), as well as between egg laying and hatching (**D**) were observed. The data represent mean ± S.E.M. (bars) (*t*-test; N = 13-15). (**E**) The cumulative percentage of females that laid eggs is displayed (Log-rank (Mantel-Cox) test; N = 13-15; p-value = 0.6993). (**F**) The number of eggs per egg pod was counted, as well as the number of hatchlings per egg pod. The data represent mean ± S.E.M. (bars), as well as the individual number of eggs (Ο) or hatchlings (x) per egg pod (*t*-test; N = 13-15).

Knockdown of *SgVKR* resulted in significantly smaller oocytes in 12-day-old adult locusts (Fig. 3A). The average oocyte size of ds*VKR*-treated animals was 4.0 mm, while the control animals had an average oocyte size of 5.1 mm. We further investigated if copulation behavior, fecundity and fertility were affected after silencing *Sg*VKR. Therefore, we observed the timing of actual copulation with a virgin male, egg laying and hatching, as well as the numbers of eggs and hatchlings. No effects of the *Sg*VKR knockdown could be observed on copulation behavior, fecundity or fertility (Fig. 3B-F).

### Effect on ecdysteroid biosynthesis

**Figure 4:**
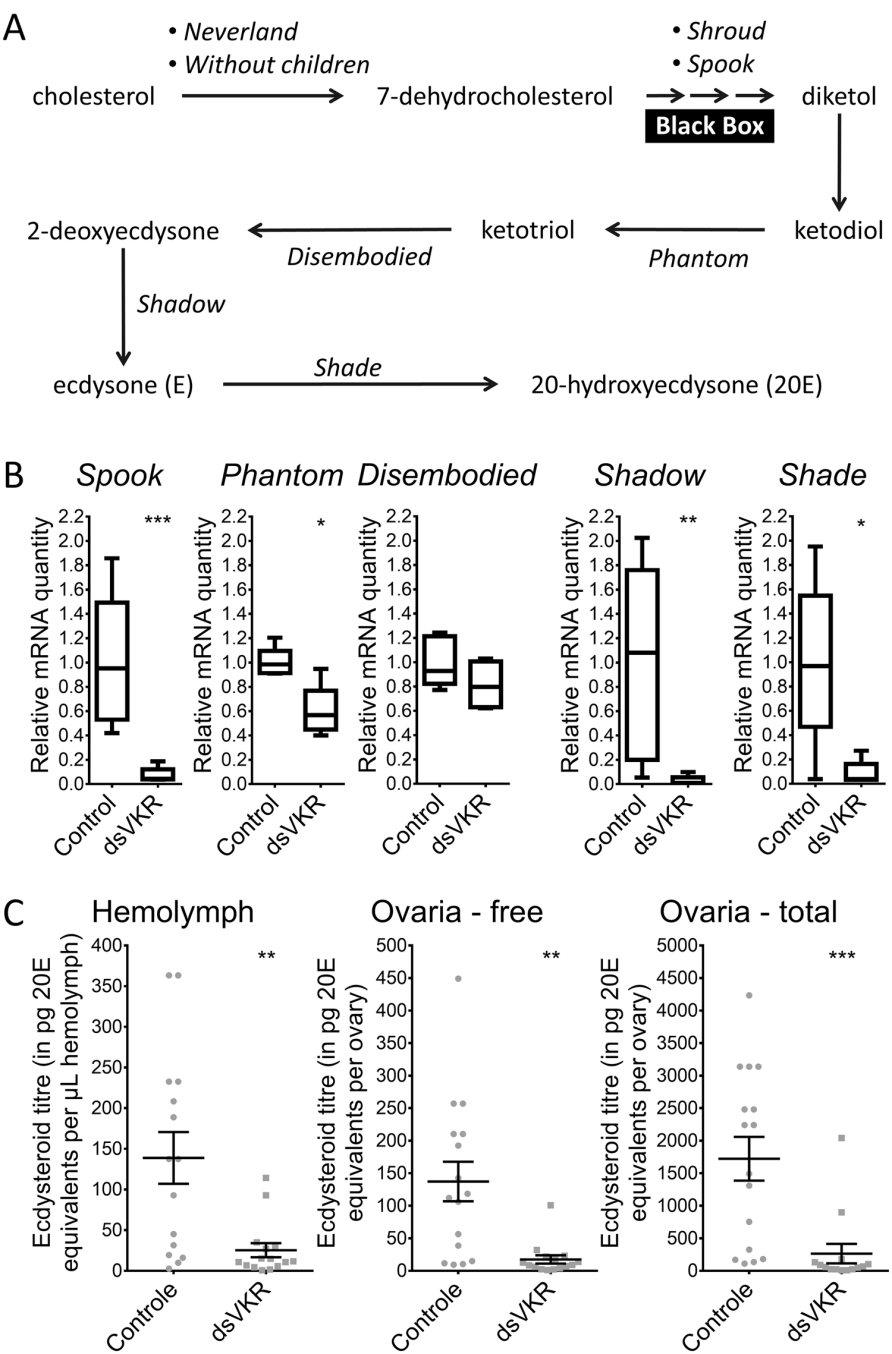
Effect of RNAi-mediated knockdown of *SgVKR* on ecdysteroid biosynthesis in 12-day-old adult female *S*. *gregaria*. (**A**) Overview of the ecdysteroid biosynthesis pathway in larval *D. melanogaster*. (**B**) Relative transcript levels of the *Halloween* genes, *SgSpo, SgPhm, SgDib, SgSad* and *SgShd*, involved in ecdysteroid biosynthesis were measured in the gonads from control and ds*VKR*-treated 12-day-old female locusts, using qRT-PCR. The data represent box plots (min to max) of five independent pools of three locusts, run in duplicate and normalized to g*lyceraldehyde-3-phosphate dehydrogenase* (*GAPDH)* and α-*tubulin1A* transcript levels. (**C**) Ecdysteroid titers in the hemolymph and ecdysteroid levels (free and total) in the gonads of 12-day-old control and ds*VKR*-treated female locusts. Ecdysteroid titers, expressed in pg 20E equivalents per µL hemolymph, and ecdysteroid levels, expressed in ng 20E equivalents per ovary, were measured with an EIA. The data represent mean ± S.E.M. of individual animals (N = 16), as well as the individual values (grey dots). Locusts were injected with 200 ng of dsRNA against *SgVKR* or *GFP* (control) one day, five days and nine days after molting to the adult stage. Significant differences (p < 0.05, p < 0.01 and p < 0.001) are indicated by (an) asterisk(s) (*, ** and *** respectively) (two-sided Welch’s *t*-test on log-transformed data).

Since silencing of *AaVKR* affected the ecdysteroid biosynthesis^5^, we investigated if this was also the case in *S. gregaria*. We first checked the transcript levels of the known *Halloween* genes, *Spook* (*SgSpo*), *Phantom* (*SgPhm*)*, Disembodied* (*SgDib*), *Shadow* (*SgSad*) and *Shade* (*SgShd*), which are involved in ecdysteroid biosynthesis by the female gonads (Fig. 4A). The relative mRNA levels of *SgSpo*, *SgPhm, SgSad* and *SgShd*, were significantly lower in 12-day-old adult females treated with dsRNA against *SgVKR* compared to control females (Fig. 4B). Most of the ecdysteroids produced by the gonads are incorporated in the growing oocytes as conjugates, while only a small fraction of the ecdysteroids is incorporated in the oocytes as ‘free’ ecdysteroids. Furthermore, a small fraction of the ovary-produced ecdysteroids will reach the hemolymph. We measured the ecdysteroid titer in the hemolymph, as well as the free and total ecdysteroid levels in the ovaries, of ds*VKR*-treated and control 12-day-old female locusts, and observed significantly lower ecdysteroid levels in the ds*VKR*-treated females compared to control females (Fig. 4C).

### Effect on expression of neuroparsins and insulin-related peptide

**Figure 5:**
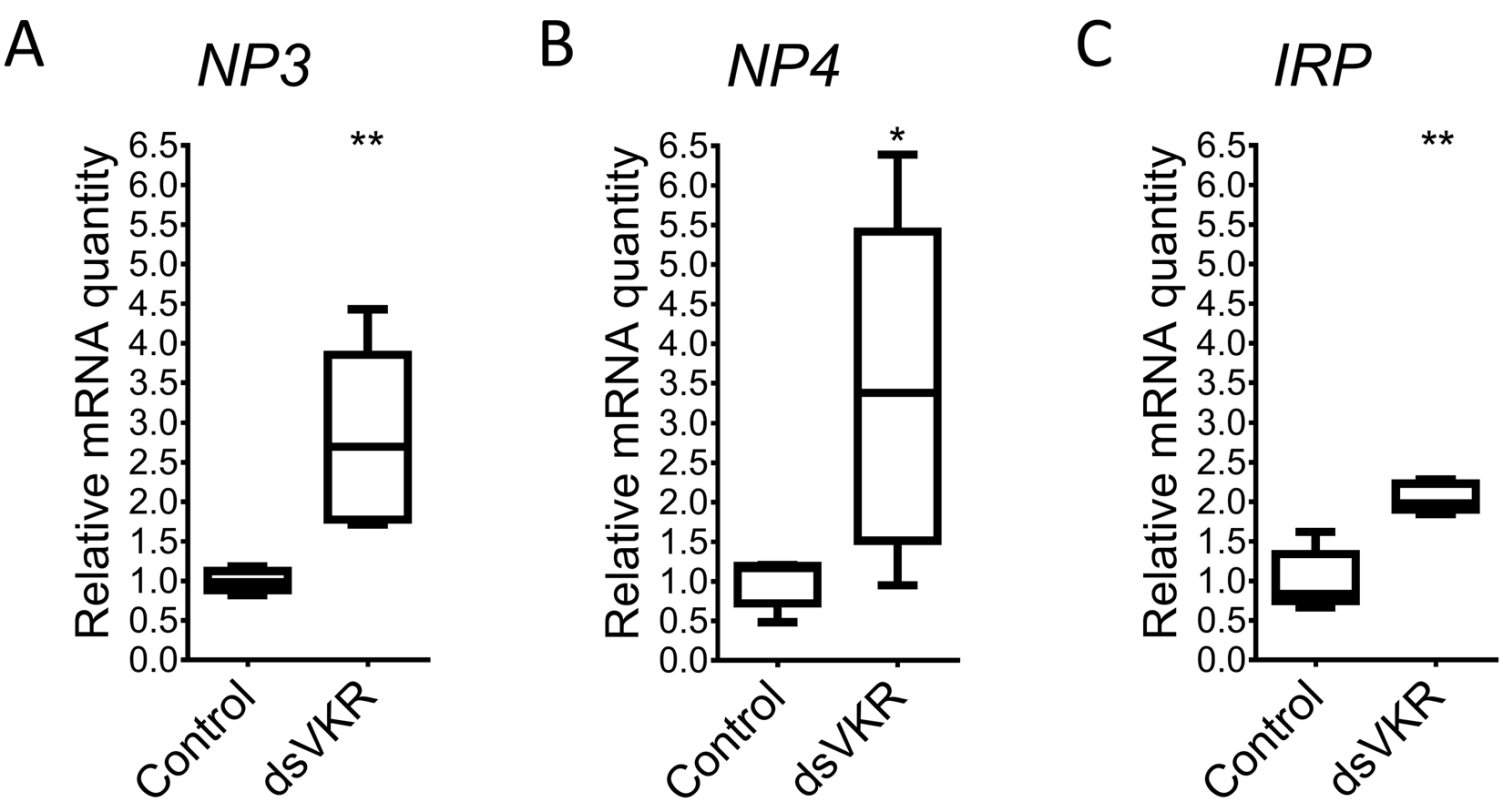
Effect of RNAi-mediated knockdown of *SgVKR* on transcripts coding for neuroparsins and insulin-related peptide in 12-day-old adult female *S. gregaria*. Relative (**A**) *SgNP3*, (**B**) *SgNP4* and (**C**) *SgIRP* transcript levels were measured in the fat body from control and ds*VKR*-treated 12-day-old female locusts, using qRT-PCR. Locusts were injected with 200 ng of dsRNA against *SgVKR* or *GFP* (control) one, five and nine days after molting to the adult stage. The data represent box plots (min to max) of five independent pools of three locusts, run in duplicate and normalized to *GAPDH* and *ribosomal protein 49* (*RP49*) transcript levels. Significant differences (p < 0.05 and p < 0.01) are indicated by (an) asterisk(s) (* and ** respectively) (two-sided Welch’s *t*-test on log-transformed data).

*In vitro* activation of *Aa*VKR by OEH, a neuroparsin (NP)-like peptide, results in phosphorylation of Ak strain transforming factor (Akt/PKB), a Ser/Thr protein kinase which also functions downstream of the insulin receptor (InR)^5^. We reasoned that if *Sg*VKR is indeed a receptor for locust NPs and/or affects processes that are also regulated by insulin-related peptide (IRP), we might observe changes in expression of NPs and/or IRP as a result of the RNAi-mediated downregulation of *SgVKR*. Our data show that silencing of *SgVKR* resulted in significantly higher relative mRNA levels of *SgNP3, SgNP4* and *SgIRP* in the fat body of 12-day-old adult female locusts (Fig. 5). When compared to control animals, neuroparsin transcripts were about 3 times upregulated, while *SgIRP* mRNA levels were *ca*. 2-fold higher in the fat body of ds*VKR*-treated animals.

### Effect on vitellogenesis and JH biosynthesis

To investigate whether the smaller oocyte size was due to reduced vitellogenin biosynthesis, we analyzed the transcript levels of the *vitellogenin 1* and *2* genes (*SgVg1* and *SgVg2*) after silencing *SgVKR* (Suppl. Figure 2A,B). Furthermore, we also checked the transcript levels of the enzymes responsible for the last two steps in juvenile hormone (JH) biosynthesis as well as the JH response gene *Krüppel-homologue 1* (*SgKr-h1*), since it is known that JH regulates vitellogenesis in *S. gregaria* (Suppl. Figure 2C-G). Although the relative mRNA levels of *SgVg1* and *SgVg2* seem lower (resp. 42% and 36%) in the fat body of 12-day-old adult females treated with ds*VKR*, they were not significantly different when statistically compared to control females. Similar results were obtained for the relative mRNA levels of the JH biosynthesis enzymes, *SgJHAMT* (*juvenile hormone acid methyltransferase*) and *SgCYP15A1* (*methyl farnesoate epoxidase*), in the CA/CC complex (resp. 34% and 40% lower on average, but not significantly). When looking at the *SgKr-h1* mRNA levels in the fat body, gonads and CA/CC complex, a reduction of 42%, 25% and 56% respectively could be calculated for ds*VKR*-treated females when compared to controls. The reduction of *SgKr-h1* transcript levels was only significantly different in the ovaries and the CA/CC complex.

## Discussion

### Characteristics of *SgVKR*

VKRs are members of the receptor tyrosine kinase (RTK) family, which are transmembrane proteins involved in many fundamental cellular processes, such as cell proliferation and differentiation^18^. Like previously identified VKRs^2,4^, the *Sg*VKR consists of a VFTM and a TK domain. The VFTM of the *Sg*VKR contains the 8 residue motif crucial for binding of amino acids (Fig. 1)^2,4^. Vanderstraete *et al*.^2^ also confirmed the requirement of the serine and arginine residues in this motif for amino acid recognition by *Am*VKR and *Sm*VKR1. These two residues are also present in the conserved motif of the VFTM of *Sg*VKR, hence a natural amino acid or derivative might be the ligand of *Sg*VKR. In spite of the presence of the serine and arginine residues in the conserved 8 residue motif in the VFTM of *Aa*VKR, this mosquito receptor was only activated upon binding of OEH^5^. However, it cannot be excluded that a small molecule was already bound to the VFT module of this receptor, as discussed by Dissous^19^. Since OEH displays sequence similarities with neuroparsins, a deducible hypothesis could be that locust neuroparsins may act as natural agonists of *Sg*VKR. An alternative hypothesis is that at some point in evolution a neuroparsin-like ligand may have been “hijacked” by a member of the VKR subfamily^20^. If so, then this evolutionary event has determined in which animal species a functional interaction between neuroparsins and VKRs is occurring. Since ligands of VKRs have only been demonstrated in *A. mellifera, A. aegypti* and *S. mansoni*, it is difficult to predict whether (an) amino acid(s) and/or (a) neuroparsin(s) can activate *Sg*VKR. The TK domain contains all motifs crucial for TK activity, such as the G-rich motif GxGxxG important in ATP binding, the VAVKx_16_E motif necessary for ATP stabilization, the HRDxAxRN motif forming the catalytic loop implicated in phosphotransfer, the triplet DGF for Mg^2+^ binding, the consensus PVRWMxPE sequence and the two putative autophosphorylation sites (YY)^2^. We can thus conclude that *Sg*VKR belongs to the RTK family and more specifically to the VKR subfamily.

### Role of *Sg*VKR in female locust reproductive physiology

VKRs play an important role in the female reproductive physiology of *S. mansoni* and *A. aegypti*, as RNAi of *SmVKR1/2* and *AaVKR* resulted in disabled egg formation^5,8^. Silencing of *AaVKR* resulted in less yolk per oocyte and lower ecdysteroid production by the ovary, because of disabled OEH activity^5^. In this mosquito, a blood meal triggers the release of OEH by the brain, which then induces the ovarian production of ecdysteroids that subsequently stimulate the fat body to produce the vitellogenins that are packaged into the oocytes. On the other hand, as in cockroaches, JH stimulates the fat body in locusts to produce vitellogenins, which will be taken up by the oocytes, while ecdysteroids produced by the ovaries are mainly incorporated into the growing terminal oocytes and are only released to a limited extent into the hemolymph towards the end of the reproductive cycle, resulting in choriogenesis and egg laying (Hult *et al*.^21^; Marchal *et al*.,^22–28^; Martín *et al*.,^23^, our unpublished data). After RNAi-mediated knockdown of *SgVKR*, we only observed smaller oocytes in 12-day-old adult female locusts (Fig. 3), but did not observe an effect on copulation behavior and post-copulation events. The fecundity or fertility of the female locusts were not affected when silencing *SgVKR*. Thus, in *S. gregaria* VKR does not seem to play a role that is as crucial as it is the case for *S. mansoni* and *A. aegypti*. It should also be noted that the locusts used in these experiments were fed *ad libitum* and kept under optimal breeding conditions. Consequently, the regulation of oocyte growth in locusts reared in these laboratory conditions might be different from the situation occurring in nature. Contrary to *A. aegypti*, where a blood meal triggers the start of oogenesis, almost like a binary switch, production of mature oocytes in locusts that occur in natural conditions may take two weeks till a few months, depending on amongst others temperature, humidity and nutrient availability^9^. Therefore, it is not because VKR does not seem to play a crucial role in these laboratory locusts, which are kept under optimal breeding conditions, that this receptor might not play a more prominent role in locusts in their natural habitat, which are highly dependent on different input signals for initiating their reproductive cycle. Furthermore, one should also keep in mind that an efficient knockdown at mRNA level does not mean that protein levels are equally lowered. Hence, there might still be enough residual VKR protein to (partially) fulfill its role, making it harder to judge the necessity of this receptor in the reproductive process.

### Cross-talk between *Sg*VKR and different hormonal pathways

This is the first report of an in-depth tissue distribution profile of *SgVKR* in insects. In *A. aegypti*, the expression of *AaVKR* was investigated in only three tissues, namely the fat body, the gut and the ovaries of female mosquitoes. While VKRs are highly expressed in the ovaries of female *S. mansoni* and *A. aegypti* ^5,8^, the relative mRNA levels of *SgVKR* are low in the locust ovaries, compared to several other tissues (Fig. 2B). Interestingly, when looking at the temporal expression profile of *SgVKR* in the female ovaries, an increase could be observed towards the end of the first reproductive cycle. This profile is similar to the temporal expression profile of two ecdysteroid biosynthesis enzymes, *SgSpo* and *SgPhm* ^15^ and correlates with the hemolymph ecdysteroid titre, suggesting this receptor could possibly regulate the ecdysteroid biosynthesis by the follicle cells surrounding the oocytes in the ovaries. When further investigating the involvement of *SgVKR* in the regulation of ecdysteroid biosynthesis, we observed that downregulation of *SgVKR* indeed resulted in lower ecdysteroid biosynthesis by the ovaries in 12-day-old adult female locusts (Fig. 4). Consequently, we hypothesize that *Sg*VKR (directly or indirectly) regulates ecdysteroid biosynthesis in adult female *S. gregaria*. This result is similar to what was found in *A. aegypti* ^5^, but probably due to the highly divergent roles of ecdysteroids in both species, silencing VKR results in a very different effect on the female reproductive physiology. Although ecdysteroid biosynthesis is affected, no effect on choriogenesis was observed. One possible explanation for this lies in the fact that we did not perform a knockout but a knockdown. In case of a knockdown, dsRNA treatment effects may be transitory. Furthermore, compensatory effects cannot be excluded.

By affecting the ecdysteroid biosynthesis *Sg*VKR appears to have a gonadotropic role, which is opposite to the anti-gonadotropic role of locust NPs^24,25^. Neuroparsin was initially discovered based on its inhibitory effects on vitellogenesis and oocyte growth, without directly affecting JH biosynthesis^25^. Recently the anti-gonadotropic role of NPs has also been confirmed in the shrimp *Metapenaeus ensis* ^26^. In *A. aegypti* on the other hand, the neuroparsin-like factor OEH has been demonstrated to act as a gonadotropic factor^7^. Based on sequence similarities with the insulin-like growth factor binding proteins (IGFBP), the hypothesis was postulated that locust NPs might act as humoral binding factors of IRP, which stimulates vitellogenesis and ecdysteroidogenesis. This hypothesis is supported by the observation of *in vitro* binding of a locust NP and IRP^27^. Therefore, locust NPs might act as negative modulators of the insulin signalling pathway, while the mosquito OEH acts in parallel with the insulin signalling pathway. Alternatively or additionally, as it is the case for several IGFBPs, NPs might also have direct functions of their own. For the latter option, one possible target could be *Sg*VKR. However, if locust neuroparsins would indeed be physiological agonists of VKR, one would expect to observe identical phenotypic effects and this is not the case^24^. On the other hand, NPs could perhaps act as allosteric inhibitors of *Sg*VKR in locusts, while the neuroparsin-like OEH acts as an activator of *Aa*VKR in mosquitoes. The latter hypothesis might also explain the opposing roles of NPs and OEH in the female reproductive physiology of respectively *S. gregaria* and *A. aegypti*. Furthermore, it should also be noted that four NPs were identified in *S. gregaria*, but their specific roles are still unknown^24^. Hence, not all NPs might act in the same manner, which would generate a much more complex situation than in several other species. Given these different hypotheses, it will be necessary to pharmacologically characterize *Sg*VKR.

One of the highest transcript levels of *SgVKR* was observed in the fat body. Since the fat body is known to be the major organ for controlling the insect’s energetic and nutritional homeostasis and for producing vitellogenins that are incorporated in maturing oocytes of adult females, it is not excluded that *Sg*VKR plays a role as a nutrient sensor or more specifically an amino acid sensor. Studies in *A. aegypti* and *S. mansoni* have proven the involvement of VKRs in the activation of the PI3K/Akt/p70S6K and ERK/ MAPK pathways, which are known to act downstream of several RTKs, such as InRs, and which are involved in the control of protein synthesis, cell growth and proliferation^5,8^. To further analyse the possible links between *Sg*VKR, *Sg*NPs and IRP/InR, we examined if silencing *SgVKR* had an influence on transcript levels of *SgNPs* and *SgIRP* (Fig. 5). The data obtained for *SgNP3*, *SgNP4* and *SgIRP* transcript levels indeed suggest that this receptor is involved in the regulation of these peptides. The smaller oocyte size observed in 12-day-old ds*VKR*-treated female locusts might perhaps be a consequence of the upregulation of *SgNP3* and *SgNP4*, since NPs are known to have an anti-gonadotropic function^24^. The upregulation of *SgIRP* may compensate for this anti-gonadotropic effect of the *Sg*NPs, but does not seem to be sufficient. These results again raise the question whether *Sg*VKR acts as a NP receptor, as discussed in the previous paragraph.

Since smaller oocytes were observed in 12-day-old *SgVKR* knockdown female locusts, we investigated if this was due to lower vitellogenin biosynthesis by the fat body. Although we repeated this experiment several times, with both dsRNA constructs, no significant differences in *SgVg1/2* transcript levels could be observed between ds*VKR*-treated and control female locusts (Suppl. Figure 2). As vitellogenesis in locusts is regulated by JHs, we also checked if the transcript levels of the last two enzymes in the JH biosynthetic pathway, *SgJHAMT* and *SgCYP15a1* ^28^, as well as the JH response gene *Kr-h1*, were altered in these ds*VKR*-treated female locusts (Suppl. Figure 2). Similarly as for *SgVg1/2* transcript, no significant differences were observed for the JH biosynthesis enzymes. However, the *Kr-h1* mRNA levels were significantly lower in the CA/CC complex and the gonads of 12-day-old ds*VKR-*treated female locusts, but not in the fat body. Based on these data, we cannot conclude that *Sg*VKR affects JH biosynthesis in the CA or vitellogenin biosynthesis in the fat body. JH-mediated signaling via Kr-h1 seems to be reduced in some tissues of the *Sg*VKR knockdown animals. These tissue-dependent effects might be caused by regulatory effects occurring downstream of JH biosynthesis and upstream of its signaling, for instance on JH degradation. The observation that *Sg*VKR knockdown does not affect *Kr-h1* transcript levels in the fat body is well in line with the data on *SgVg1/2* expression. On the other hand, it should also be noted that the knockdown of *SgVKR* in the Fb and CA/CC complex was not as potent as in the ovaries. As such, it is possible that this resulted in absence of significant differences in fertility or in transcript levels of the vitellogenins and JH biosynthesis enzymes, when compared to the respective control conditions. Furthermore, it should also be noted that the dsRNA treatment in *S. gregaria* generates a systemic knockdown, so it is currently difficult to make a distinction between the direct and indirect *in vivo* roles of *SgVKR*.

## Conclusion

We cloned the *Sg*VKR from *S. gregaria* and investigated its *in vivo* role. By means of RNAi, we have shown a gonadotropic function for this receptor in adult female locusts. However, this locust VKR did not seem to be as crucial as the VKRs in *S. mansoni* and *A. aegypti* for controlling reproductive success in adult females. While *Sg*VKR knockdown, as in *A. aegypti*, significantly reduced ovarian ecdysteroid biosynthesis, only a limited effect was observed on oocyte size and fertility was not affected. Although its mode of action is not yet entirely clarified, *Sg*VKR does not show the same phenotypic effects as locust NPs. It is possible that *Sg*VKR acts as a nutrient-dependent sensor in several tissues, where it may contribute to the complex coordination by different pathways that regulate the process of egg production in adult female *S. gregaria*.

## Electronic supplementary material

Supplementary figures
