# Supplementary material for: Role of the venus kinase receptor in the female reproductive physiology of the desert locust, Schistocerca gregaria
Source: Sci Rep. 2017 Sep 15;7:11730. doi: 10.1038/s41598-017-11434-3 (PMC5601475; doi:10.1038/s41598-017-11434-3)
Supplement: Supplementary file 1 — Supplementary figures [file 41598_2017_11434_MOESM1_ESM.pdf]

Role of the venus kinase receptor in the female reproductive physiology of the desert locust, *Schistocerca gregaria.*

Cynthia Lenaerts[1], Jolien Palmans[1], Elisabeth Marchal[1], Rik Verdonck[1], Jozef Vanden Broeck[1]*

Supplementary figures

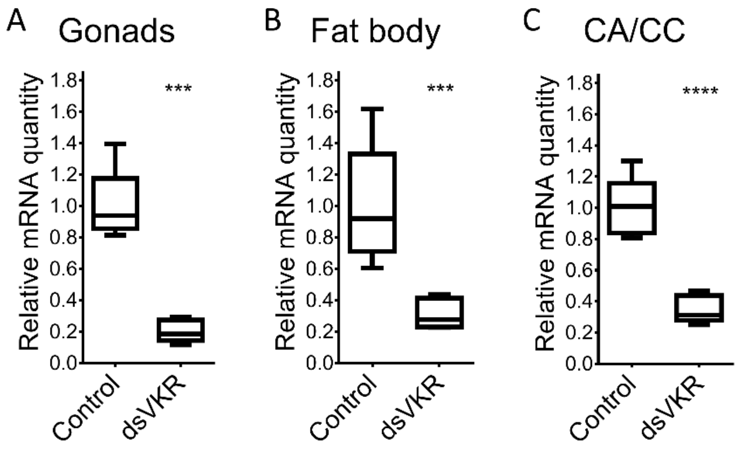

Supplementary figure S1. Efficiency of the RNAi-mediated knockdown of *SgVKR* in 12-day-old adult female *S. gregaria*. Relative *SgVKR* transcript levels were measured in (A) the gonads, (B) the fat body and (C) the CA/CC complex from control and ds*VKR*-treated 12-day-old female locusts, using qRT-PCR. Locusts were injected with 200 ng of dsRNA against *SgVKR* or *GFP* (control) one, five and nine days after molting to the adult stage. The data represent box plots (min to max) of five independent pools of three locusts, run in duplicate and normalized to *GAPDH* and α-*tubulin1A* transcript levels for the gonads, *GAPDH* and *RP49* transcript levels for the fat body, and *β-actin* and *elongation factor 1α* (*EF1α*) transcript levels for the CA/CC. Significant differences ($p < 0.01$ and $p < 0.001$) are indicated by asterisks (** and *** respectively) (one-sided Welch's *t*-test on log-transformed data).

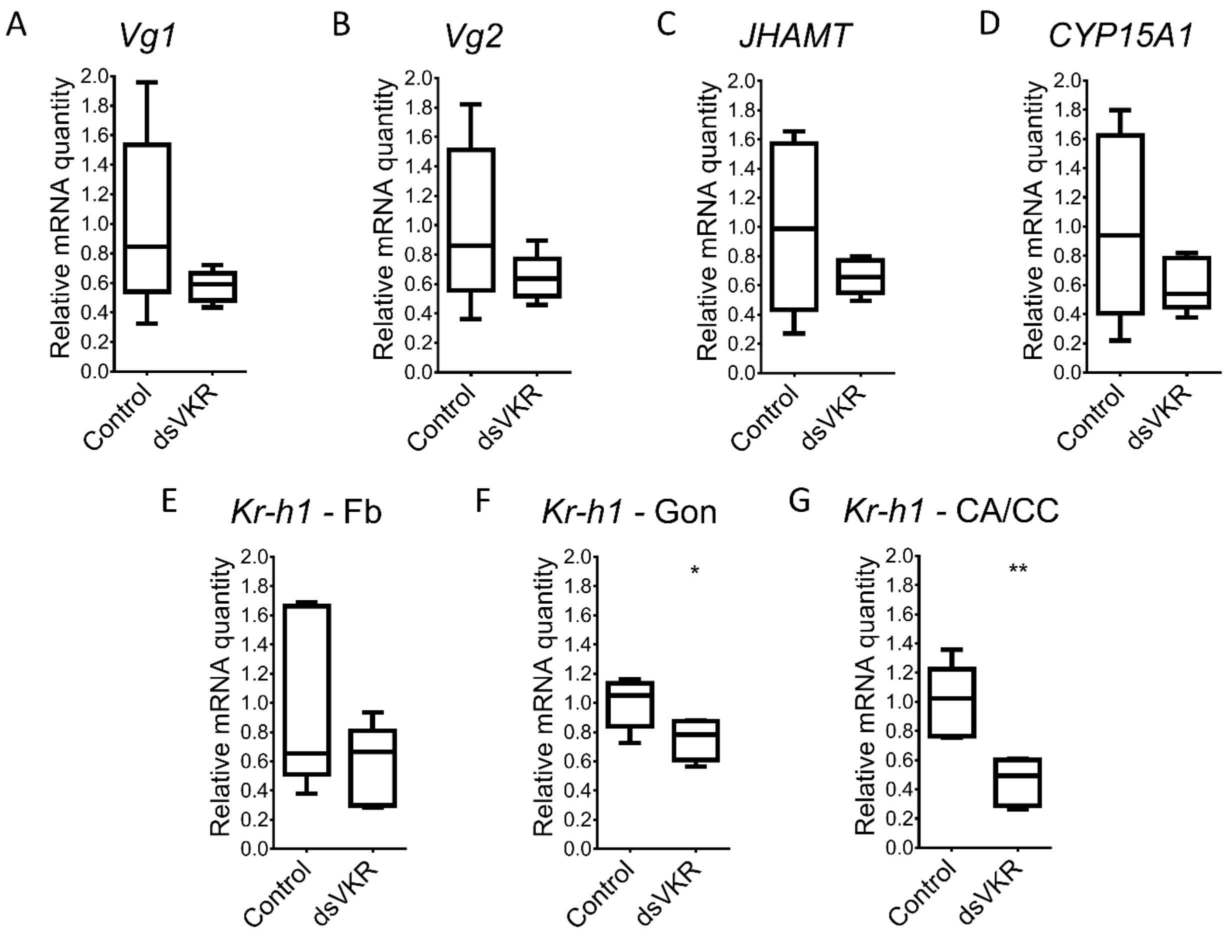

Supplementary figure S2. Effect of RNAi-mediated knockdown of *SgVKR* on vitellogenesis and JH synthesis in 12-day-old adult female *S. gregaria*. (A-B) Relative *SgVg1* and *SgVg2* transcript levels were measured in the fat body from control and ds*VKR*-treated locusts, using qRT-PCR. (C-D) Relative transcript levels for the last two enzymes involved in the synthesis of JH (*SgJHAMT* and *SgCYP15A1*) were measured in the CA/CC complex from control and ds*VKR*-treated locusts, using qRT-PCR. (E-G) Relative transcript levels of the JH response gene, *SgKr-h1*, were measured in the (E) fat body, (F) gonads and (G) CA/CC complex from control and ds*VKR*-treated locusts, using qRT-PCR. Locusts were injected with 200 ng of dsRNA against *SgVKR* or *GFP* (control) one, five and nine days after molting to the adult stage. The data represent box plots (min to max) of five independent pools of three locusts, run in duplicate and normalized to g*lyceraldehyde-3-phosphate dehydrogenase* (*GAPDH)* and α-*tubulin1A* transcript levels for the gonads, *GAPDH* and *ribosomal protein 49* (*RP49*) transcript levels for the fat body, and *β-actin* and *elongation factor 1α* (*EF1α*) transcript levels for the CA/CC. Significant differences ($p < 0.05$ and $p < 0.01$) are indicated by (an) asterisk(s) (* and ** respectively) (two-sided Welch's *t*-test on log-transformed data).
